# Functional Characteristic and Significance of Aldosterone-Producing Cell Clusters in Primary Aldosteronism and Age-Related Hypertension

**DOI:** 10.3389/fendo.2021.631848

**Published:** 2021-03-03

**Authors:** Fatin Athirah Pauzi, Elena Aisha Azizan

**Affiliations:** Department of Medicine, Faculty of Medicine, Universiti Kebangsaan Malaysia Medical Centre, Cheras, Malaysia

**Keywords:** primary aldosteronism, aldosterone, adrenal, aldosterone-producing cell clusters, hypertension

## Abstract

Primary aldosteronism (PA) is one of the most frequent curable forms of secondary hypertension. It can be caused by the overproduction of aldosterone in one or both adrenal glands. The most common subtypes of PA are unilateral aldosterone over-production due to aldosterone-producing adenomas (APA) or bilateral aldosterone over-production due to bilateral hyperaldosteronism (BHA). Utilizing the immunohistochemical (IHC) detection of aldosterone synthase (CYP11B2) has allowed the identification of aldosterone-producing cell clusters (APCCs) with unique focal localization positive for CYP11B2 expression in the subcapsular portion of the human adult adrenal cortex. The presence of CYP11B2 supports that synthesis of aldosterone can occur in these cell clusters and therefore might contribute to hyperaldosteronism. However, the significance of the steroidogenic properties of APCCs especially in regards to PA remains unclear. Herein, we review the available evidence on the presence of APCCs in normal adrenals and adrenal tissues adjacent to APAs, their aldosterone-stimulating somatic gene mutations, and their accumulation during the ageing process; raising the possibility that APCCs may play a role in the development of PA and age-related hypertension.

## Introduction

Arterial hypertension is a worldwide health problem that affects approximately 1.13 billion of the global population (1) and is estimated to cause 7.5 million deaths or approximately 12.8% of the total all deaths (2). In more than 90% of patients, there is not a single cause for arterial hypertension, known as essential or primary hypertension (3). However, in certain cases, hypertension may emerge from a specific disease and therefore known as secondary hypertension, which includes endocrine hypertension that develops following a dysregulation of one or more hormones that are implicated in blood pressure regulation (4). Primary aldosteronism (PA), also known as Conn’s syndrome, is one of the most frequent curable forms of secondary/endocrine hypertension (5). This syndrome accounts for 7–10% of all referred hypertensive patients (6–8), 4% in primary care (9), and 15–20% of patients with resistant hypertension (10, 11). PA is caused by the autonomous aldosterone production in one or both adrenal glands, in which patients with PA are clinically associated with high levels of aldosterone despite being under suppressed renin conditions (12).

Under physiological conditions, the two primary systems that regulate aldosterone production are the renin-angiotensin-aldosterone system (RAAS) and the hypothalamic-pituitary-adrenal axis, in addition to blood potassium levels (13, 14). However, in PA, excess aldosterone independent of the RAAS acts on the distal tubule and medullary collecting duct to increase sodium reabsorption as well as potassium excretion (13). Water reabsorption follows salt, causing volume expansion that develops into hypertension and in some cases hypokalemic metabolic alkalosis (14). Hence, clinical presentation for hypokalemia, though not specific for PA, may also present as cramping and muscle weakness, palpitations, polydipsia, and polyuria among others (13). Furthermore, chronic inappropriate elevations of aldosterone levels in PA patients can increase pro-inflammatory cytokines (15), causing oxidative stress (16), which in the long-term leads to tissue damage and fibrosis (17). Compared to the age-, sex-, and blood pressure-matched essential hypertension patients, the risk for cardiovascular complications is significantly higher in PA patients (18, 19). This suggests the impact of autonomous aldosterone production on cardiovascular complications is beyond the increase in blood pressure (20–22).

Due to the high prevalence of PA among hypertensive patients and the increased risk for cardiovascular complications, early diagnosis as well as targeted treatment is of importance (12). The biochemical picture of patients with PA comprises of high aldosterone to renin ratio which has become one of the primary PA’s screening tools alongside different confirmatory tests, that includes fludrocortisone suppression test, intravenous saline infusion test, oral salt-loading test, or captopril test, which are all to be performed after a positive screening test (i.e. high aldosterone to renin ratio) (13, 18). The current guidelines recommend that when aldosterone is overproduced unilaterally, as detected by adrenal vein sampling (AVS), or in some exceptions when CT-scan detects a unilateral adenoma in a young hypokalemic PA patient (<35 years old), unilateral adrenalectomy is the treatment of choice. Conversely, mineralocorticoid receptor antagonists are utilized when AVS implies bilateral hyperaldosteronism or when the patient is not suitable for surgery (18). As identifying the most suitable therapy for PA is heavily influenced by the laterality of aldosterone overproduction, AVS, the gold standard to determine unilateralization (13), should be used where possible.

The majority of unilateral PA is due to an aldosterone-producing adenoma (APA), whereas bilateral PA, also known as bilateral hyperaldosteronism (BHA), are mostly due to adrenal zona glomerulosa hyperplasia or bilateral micronodular hyperplasia (23, 24). Bilateral PA can be further categorized into CT-negative idiopathic hyperaldosteronism (IHA) and rarely CT-positive bilateral APA (25–27). Approximately, APA and BHA account for 35%–40% and 60% of PA cases, respectively (28). In addition, there are less common subtypes of PA that include adrenal carcinoma, rare familial subtypes, and unilateral adrenal hyperplasia (29). It is postulated that bilateral PA could be caused by the accumulation of aldosterone-producing cell clusters (APCCs) (23), i.e. clusters of cells autonomously expressing aldosterone synthase (CYP11B2). Interestingly, these APCCs are not only found in the cells adjacent to an APA of which excised tissue is available (24, 30–33) but also in normal adrenal glands (30, 34, 35), with the prevalence seeming to increase with age (34–36). Hence, it is of importance to understand whether these clusters could contribute to the state of hyperaldosteronism or affect the increased prevalence of hypertension occurring with age.

## Characteristics of Aldosterone-Producing Cell Clusters (APCCs)

Physiologically, aldosterone synthesis occurs in the most outer zone of the adrenal, the zona glomerulosa (ZG) (23). This is because the cells in this zone can express CYP11B2, the enzyme aldosterone synthase, which is essential for the final steps of aldosterone synthesis. The synthesis of aldosterone is primarily stimulated by the activation of intracellular calcium signaling in the ZG that can be mediated either by angiotensin II from the renin-angiotensin system or by extracellular potassium levels. Similar to aldosterone, adrenal cortisol steroid production physiologically occurs in distinct zones of the adrenal cortex, the zona fasciculata (ZF), due to the expression of 11β-hydroxylase (CYP11B1), the enzyme responsible for the final reaction in cortisol production. The synthesis of cortisol is primarily regulated by the hypothalamus-pituitary-adrenal axis mainly through the adrenocorticotropic hormone (ACTH) (4). Hence, in conventional adrenal zonation, CYP11B2 is only expressed in the ZG but not in ZF, whereas CYP11B1 is expressed in the ZF but not in the ZG (30, 37–40). However, immunohistochemical characterization of the adrenal gland by Nishimoto et al. (30) has led to the novel discovery of microscopic clusters of subcapsular adrenal cells that express CYP11B2 that extends from the ZG into the ZF which have been termed as APCCs. However, as the term “cell cluster” in APCCs does not describe the histology and pathology of the feature a recent consensus on histopathology of PA adrenals recommends the usage of the term “aldosterone-producing micronodule” instead of APCCs (41).

Nevertheless, whether termed APCCs or aldosterone-producing micronodule, the definition of this feature varies between research groups and a clear description that can distinguish this unique structure from APA and adjacent normal ZG cells has yet to be established. The size of APCCs has been reported to be smaller, approximately 0.2–1.5 mm in length, than APAs that are typically more than 3 mm in length (30, 36, 42). Further, APCCs have been characterized as having non-adenomatous features unlike that seen frequently with APA, e.g. not having a fibrous capsule or having cellular/tissue atypia (12). APCCs have also been described to be composed of morphological ZG cells in contact with the capsule and inner columnar ZF like cells forming cords along sinusoids (30). The current common definition for APCCs is to have positive CYP11B2 expression in a unique focal localization (30). As the morphology of APCC cells appears to be remarkably close to those of non-APCCs (33, 35), they cannot be easily distinguished from adjacent normal cortical cells by routine hematoxylin and eosin staining (30, 36, 42). Instead, immunohistochemistry (IHC) of CYP11B2 has been the method of choice to identify the presence of APCCs (30, 43).

IHC analysis has shown that compared to ZF cells that are generally negative for CYP11B2 and unrelated to aldosterone production, ZF-like looking cells of APCCs in the ZF has high levels of CYP11B2 and low levels of CYP11B1 or CYP17A1, both enzymes being involved in cortisol production (33, 35, 37, 38). However, according to Lim & Rainey (44), APCCs may manifest various patterns of enzyme expression with some exhibiting homogeneous expression of CYP11B2 while others exhibit polarity with a declining expression of CYP11B2 aligned with the development of CYP17A1 expression. This pattern of expression indicates that some APCCs cells may go through a transition to a more ZF-like phenotype, at least partially (44), thus making CYP11B1 and CYP17A1 IHC useful to characterize APCCs (33, 35). Another published method to detect APCCs is *in situ* hybridization of CYP11B2. Utilizing this method, Boulkroun et al. (45) distinguished three CYP11B2-expressing structures beneath the adrenal capsule that were termed as foci, megafoci and APCCs based on the size of cell clusters and their relative expression of a glomerulosa marker, Disable-2 (DAB2). In this study, APCCs were described as large clusters of CYP11B2 cells that did not express DAB2 indicating that APCCs contain cells presenting with an intermediate phenotype between ZG and ZF cells (45). However, on the transcriptomic level, Omata et al. (12) reported APCCs to be more similar to the adrenal ZG transcriptome than ZF or zona reticularis transcriptomes.

## Physiological and Pathological Aspects of APCCs

APCCs are now accepted as a common feature in adrenal tissues adjacent to an APA (24, 30–33). Analysis of the adrenal glands of PA patients demonstrated that 40% to 50% of adrenals with APAs contained APCCs in the non-tumor portions of APA (30, 31). Interestingly, in Kometani et al. (32) study that found a 94% prevalence of APCCs in 16 adrenal tissue adjacent to the APA; the number and summed-area of APCCs in APAs were significantly higher in patients with discordant AVS results whose diagnosis changed to bilateral PA post-adrenocorticotropic hormone (ACTH) stimulation. The authors therefore proposed that APA patients with multiple APCCs might have similar adrenocortical pathological conditions in the contralateral adrenal gland. This assumption would explain their finding that in the pre-ACTH group having multiple APCCs, the post- to pre-ACTH plasma aldosterone concentrations (PACs) ratio was markedly higher on the non-dominant than the dominant side. Although the aldosterone responsiveness of APCCs towards ACTH has not been thoroughly described, it is conceivable that ACTH stimulation triggers the synthesis of aldosterone by APCCs in the contralateral adrenal gland, resulting in a decreased lateralization index (32). As RAAS is systemically suppressed in PA patients, these findings indicate that APCCs might play a role in autonomous aldosterone production (12). Concurringly, APCCs were also reported to be increased in adrenals of unilateral CT-negative PA compared to normotensive adrenals, implying that the elevated number of adrenal CYP11B2-expressing nodules might be the cause of hyperaldosteronism in these patients (33). Moreover, in one study that investigated the presence of APCCs in adrenals from IHA PA patients, all IHA adrenals were found to have at least one APCC or a microAPA (24). The number of APCCs in IHA adrenals were markedly larger compared to the cohorts of age-matched normotensive adrenals (24). Thus, these cell clusters could be the cause of hyperaldosteronism in these patients. This was proved to be the case in four patients with unilateral APCCs (aldosterone-producing micronodules not more than 3 mm in greatest dimension) whereby unilateral adrenalectomy resulted in a complete amelioration of blood pressure and hormonal abnormalities (46). Due to the small sizes of APCCs, patients with this feature may often be misdiagnosed as IHA as standard imaging test may not identify tiny anomalies of adrenals. Thus AVS, or if possible segmental selective AVS, should be used as part of the diagnostic procedure (47).

On the other hand, APCCs have not only been found in pathological adrenal tissues but also in normal adrenals where no tumors were present (30, 34, 35). In situ hybridization studies have found that APCCs in normal adrenals have a very similar profile to APCCs found in adrenal tissue adjacent to an APA (48, 49). In a study of nine Japanese patients with renal cell carcinoma (RCC) or upper urinary tract urothelial carcinoma (*n*=1), the presence of APCCs was found in eight of nine normal adrenals (30). Concurringly, another study from Japan on normotensive adrenal glands procured from an autopsy cohort found that out of 107 adrenals, 61 APCCs were detected in 31 autopsy cases (35). Similarly, a larger study by Nanba et al. (34) that analyzed the relationship between age and adrenal CYP11B2 expression, reported 69% of normal adrenal glands (88/127) had at least one APCC. Interestingly, this study also reported that although the total CYP11B2-expressing area negatively correlated with age (r=-0.431, P<0.0001), the total APCCs area positively correlated with age (r=0.390, P<0.0001) (34).

## Significance of APCCs to Age-Related Hypertension

Owing to the fact that APCCs are frequently seen in non-hypertensive as well as pathological human adrenal glands expressing CYP11B2, several studies have investigated if these so-called APCCs detected through CYP11B2 expression could produce aldosterone. By utilizing *in situ* hybridization method, Shigematsu et al. (49) reported the presence of APCC-like subcapsular micronodules, showing intense transcript expression for HSD3B2, CYP11B1, and CYP11B2, but not CYP17A1, implying that the nodules have the steroidogenic enzymatic property needed to synthesize aldosterone and thus might be responsible for hyperaldosteronism. This was supported by Sugiura et al. (50) study of PA adrenal sections using Fourier transform ion cyclotron resonance mass spectrometry (FT-ICR-MS) and tandem mass spectrometry imaging that demonstrated high levels of aldosterone and 18-oxocortisol, a potential serum marker of APA, in APCCs. Similarly, a recent study using tandem mass spectrometry imaging verified Suguira et al’s findings in normal adrenals as elevated levels of aldosterone and 18-oxocortisol were found in APCC’s relative to the adjacent adrenal tissue in an adrenal obtained from a patient that had undergone radical nephrectomy for renal cell carcinoma (51). These studies support the steroidogenic activity of APCCs in producing aldosterone in normal adrenals as well as under suppressed renin conditions, as shown in PA patients’ adrenals (44). Accordingly, APCCs has been proposed to be the transitioning step to APAs (50).

Based on aldosterone studies from their groups and others, Sugiura et al. (50) suggested that with ageing, APCCs accumulate aldosterone-driver mutations causing autonomous aldosterone production that can lead to APCC-to-APA translational lesions (pAATL). This is as with age, there is a loss of the classical subcapsular CYP11B2-positive ZG due to the effects of trophic hormones that lead to the decline in total aldosterone production. Thus within this background of decreased ZG CYP11B2 expression, the number of CYP11B2-positive APCCs grows (34, 44). Finally, the expansion of the mutation bearing APCCs leads to the generation of an aldosterone-producing adrenal lesion. The age-related progressive pattern of APCCs has been validated by several studies that showed the number of adrenal APCCs increased with ageing in normal adrenals from kidney donors (34) and non-hypertensive Japanese cohorts (33), signifying the contribution of APCCs in the early stages of PA in ageing adults. Altogether, the findings of APCCs in normal adrenals and the adrenal glands of PA patients with APAs, as well as their accumulation during the ageing process, has brought up the possibility that APCCs might play a role in the development of APAs and therefore is a continuum of PA.

## Somatic Gene Mutations of APCCs

Whole exome sequencing studies on APAs have defined recurrent somatic mutations in genes coding for ion channels (*KCNJ5*, *CACNA1H*, and *CACNA1D*) and ATPases (*ATP1A1* and *ATP2B3*) that either caused cytosolic acidification (for *ATP1A1*) or activated intracellular calcium signaling by depolarization of ZG cell membrane that opens the voltage-gated calcium channels or by affecting the intracellular calcium recycling (4, 52–56). This ultimately results in an increase of CYP11B2 expression that leads to aldosterone overproduction. The promising discovery of recurrent somatic mutations in APAs through the application of next-generation sequencing (52–55) has driven several studies to utilize this platform to determine the prevalence of aldosterone-driving somatic mutations in APCCs. From a cohort of 42 normal adrenals among kidney donors, 8 of 23 APCCs (35%) were found to harbor known APA-associated driver mutations, predominantly in *CACNA1D* (26%) followed by *ATP1A1* (9%) (43). However, this study did not find any APCCs to harbor the *KCNJ5* mutations, the most common mutations found in APAs (43). Similarly, in another study that performed next-generation sequencing analysis on CYP11B2-expressing nodules from the adrenals of PA patients that were CT-negative for adenomas, APCC-like micronodules were found to pre-dominantly harbor *CACNAID* somatic mutations (33).

Compared to *KCNJ5* mutant APAs that have been described as more prevalent in young women with higher levels of plasma aldosterone and larger ZF-like APAs (12, 57–60), *CACNA1D* mutations are associated with smaller ZG-like APAs in older men that are more difficult to be diagnosis (43, 53, 60). *CACNA1D* encodes for the voltage-dependent L-type (long-lasting) calcium channel subunit alpha-1D (the Cav1.3 calcium channel) (52, 53). This calcium channel is composed of 4 homologous repeated domains with 6 transmembrane segments (S1-S6) each and a membrane-associated loop between S5 and S6 (52, 53, 61). Mutations occurring in *CACNA1D* are gain of function mutations that lead to a shift of the voltage-dependent channel activation to more negative voltages or delay the inactivation of the channel. The resulting net effect is increased intracellular calcium concentrations and thereby induction of excessive aldosterone synthesis (52, 53). Hence, the high prevalence of APCCs with *CACNA1D* mutations suggests that an intracellular increase of calcium is causal for the high CYP11B2 expression in APCCs (62).

A study on 107 adrenal glands of normotensive Japanese patients from an 837 consecutive autopsy cohort was conducted to test the hypothesis that hyperaldosteronism in the group of CT-negative PA patients was induced by the elevated number of adrenal CYP11B2-expressing nodules (33, 35). The study in adrenals from non-hypertensive Japanese patients demonstrated that APCCs were common with 34% harboring known aldosterone-driver somatic mutations, *CACNA1D* being the most frequent mutations while there being no detections of *KCNJ5* mutant APCCs (35). The mutations were found to be present in both the ZG and ZF-like components of APCCs and absent in neighboring cells negative for CYP11B2 (35). The similarity of APCCs somatic mutation spectrum observed in this normotensive cohort to that found in PA patients that had adrenals that were CT-negative for adenomas implied that normal adrenal glands may progress to PA through an increase in the APCCs frequency (12). Concurringly, a study on the IHA subtype of PA found that in 15 IHA adrenals, 99 APCCs were detected of which 58% had a *CACNA1D* mutation thus supporting the notion that PA in IHA may result from the accumulation or enlargement of CT-undetectable APCCs harboring APA-associated somatic mutations that increases aldosterone production (24).

The high presence of aldosterone-driver mutations in APCCs, especially in *CACNA1D* and *ATP1A1* (as shown in **Table 1**), supports the suggestion that APCCs are capable of autonomous aldosterone production and therefore could be the origin of APAs. Nishimoto et al. (42, 43) proposed that the series of events contributing to emergence of APAs from APCCs arises through the formation of possible APCC-to-APA transitional lesions (pAATLs) that has been characterized to consist of an outer APCC-like portion and an inner micro-APA (mAPA)-like portion (42). Interestingly, in one pAATL, the same *ATP1A1* mutation has been found in the APCC-like portion and the mAPA-like portion of the pAATL, indicating that these portions have a clonal origin (42). However, the genetic characteristic of pAATL is heterogeneous. In one adrenal gland, Nishimoto et al. (42) found the mAPA-like portions of pAATLs examined harbored *KCNJ5* mutations whereas their corresponding APCC-like portions did not, with the mutation being different from that identified in the APA within the same adrenal. This indicates that the APA and the pAATL had different origins and that the mAPA-like portion arises from existing APCCs due to the introduction of a *KCNJ5* mutation that differentiated the mAPA portion from the APCC. The lack of detection of *KCNJ5* mutation in the APCC-like portion could be due to a rapid progression to APA, therefore hard to be witnessed prior to its development (43, 63). However, some authors postulate that *KCNJ5* mutation could rather be a second hit mutation (than a causal mutation of pAATL), as only pAATL nodules larger than 3 mm have been reported to harbor *KCNJ5* mutations (63). Thus, these observational findings require further functional analysis to better elucidate the genomic events involved in the transition of APCCs to APA.

**Table 1:** Somatic gene mutations detected in APCCs.

Gene	Mutation	Reference
*ATP1A1*	L104R*	(33)
	L104V*	(40)
	V332G*	(40)
	E687K	(33)
	M734L	(40)
*ATP2B3*	D77N	(33)
	E135K	(33)
	G270D	(33)
	G325D	(33)
	R345Q	(40)
	A790V	(33)
	S1137F	(33)
	P1150L	(33)
	A1157V	(33)
*CACNA1D*	E124K	(30)
	L248F	(30)
	V259G	(30)
	L272R	(30)
	G323R	(30)
	V401L*	(30)
	G403R**	(30, 33, 40)
	S410L	(30, 33)
	G457R	(33)
	R510X	(33)
	P548L	(33)
	L613Q	(40)
	R619W	(40)
	S652L*	(30)
	L653P	(30)
	S724L	(30)
	F747C	(33)
	F747L**	(30, 33, 40)
	F747V**	(30, 33, 40)
	L748S	(30)
	755_757del	(30)
	S969L	(30)
	R990H**	(30, 33)
	A998V**	(30, 33)
	A1011T	(30)
	I1015V	(30)
	F1147C	(33)
	F1147L	(30, 33)
	R1183H	(30)
	F1248L	(30, 33)
	D1273N	(30)
	P1336R*	(33)
	V1338M**	(30, 33, 40)
	I1352T	(33)
	P1499L	(33)
	T1835I	(30)
	W1836X	(33)

*Mutations that have been previously reported in sporadic APAs.

**Mutations that have been previously reported in APAs and functionally characterized.

## Conclusions

In conclusion, several studies have found the presence of aldosterone-driving somatic gene mutations, similar to those found in APAs, among APCCs which supports the autonomous secretion of aldosterone by these clusters of cells and its role in the pathology of PA. The high frequency of *CACNA1D* and *ATP1A1* mutations, but not *KCNJ5* mutations in APCCs, suggest that these cells could be a potential precursor for ZG-like APAs rather than ZF-like APAs. Further, there is supporting evidence to suggest that accumulation of APCCs with age might contribute to the increased prevalence of hypertension occurring with age. More studies on APCCs are necessary to clarify the pathological mechanisms (and perhaps physiological as also found in “normal” adrenals) in regulating aldosterone production in these clusters of cells. As technology develops, whole-genome sequencing may be the important step forward to reveal novel gene mutations that lead to the development of aldosterone-driving somatic mutation. Identifications of germline variants may help to elucidate the potential mechanisms and pathways that lead to accumulation of aldosterone-driving somatic mutations and thus may consequently provide novel personalized treatment for age-related hypertension.

## Author Contributions

EA and FP conceived the presented idea. EA supervised and verified the concept. FP prepared the original draft. EA and FP reviewed and edited the manuscript. All authors contributed to the article and approved the submitted version.

## Funding

FP is supported by the program Malaysia Partnership and Alliances in Research (MyPAiR) Newton-MRC UK-MY (NEWTON-MRC/2020/002). EA is supported by the Royal Society-Newton Advanced Fellowship (FF-2018-033/NA170257).

## Conflict of Interest

The authors declare that the research was conducted in the absence of any commercial or financial relationships that could be construed as a potential conflict of interest.
